# Preoperative neutrophil to lymphocyte ratio improves recurrence prediction of non-muscle invasive bladder cancer

**DOI:** 10.1186/s12894-018-0404-x

**Published:** 2018-10-22

**Authors:** Itamar Getzler, Zaher Bahouth, Ofer Nativ, Jacob Rubinstein, Sarel Halachmi

**Affiliations:** 1 Department of Urology, Bnai Zion Medical Center, Faculty of Medicine, Technion - Israel Institute of Technology, Golomb 47, 31048 Haifa, Israel; 2 Department of Mathematics, Technion - Israel Institute of Technology, Haifa, Israel

**Keywords:** Neutrophil-lymphocyte ratio, NLR, NMIBC, urothelial carcinoma, Recurrence, Bladder cancer

## Abstract

**Background:**

This study aims to prospectively evaluate the ability of Neutrophil-to-Lymphocyte ratio (NLR) to forecast recurrence in patients with non-muscle invasive bladder cancer (NMIBC). This is a continuation of our two previous retrospective studies that indicated the NLR > 2.5 criterion as a predictor of recurrence in patients with NMIBC.

**Methods:**

Since December 2013, all patients admitted to our department for TUR-BT and agreed to participate, had a blood drawn for cell count and differential 24 h prior to surgery. Patients with pathological NMIBC were followed prospectively for disease recurrence. The end-point of the follow up was either a cancer recurrence or the termination of the study. Univariate and multivariate Cox regressions were performed to assess the NLR > 2.5 predictive capability for recurrence, versus and in conjunction to the pathologically based EORTC score, among additional statistical analyses.

**Results:**

The study cohort included 96 men and 17 women with a median age of 72 years. Sixty-four patients (56.6%) have had a recurrence during the study occurring at the median time of 9 months (IQR 6, 13), while the median follow-up time for patients without recurrence was 18 months (IQR 10, 29). Univariate Cox regressions for recurrence demonstrated significance for NLR > 2.5 for the whole cohort (*p* = 0.011, HR 2.015, CI 1.175–3.454) and for the BCG sub-group (*p* = 0.023, HR 3.7, CI 1.2–11.9), while the EORTC score demonstrated significance for the ‘No Treatment’ subgroup (*p* = 0.024, HR 1.278, CI 1.03–1.58). When analyzed together as a multivariate Cox model, the NLR > 2.5 and EORTC score retained their significance for the aforementioned groups, while also improving the EORTC score significance for the whole cohort.

**Conclusion:**

NLR > 2.5 was found to be a significant predictor of disease recurrence and demonstrated high hazard ratio and worse recurrence-free survival in patients with NMIBC, especially in those treated with BCG. Additionally, our data demonstrated statistical evidence that NLR > 2.5 might have an improving effect on the EORTC score’s prediction when analyzed together.

## Background

Bladder cancer is the most common malignancy of the urinary tract, and the 4th most common cancer in males in developed countries [1]. Upon diagnosis, the majority (~ 75%) of patients with bladder cancer present with non-muscle invasive disease (NMIBC), which by definition includes the Tis, Ta and T1 pathologic stages [2]. As such, NMIBC represents a heterogeneous group of tumors with different rates of recurrence, progression and disease-related mortality. Consequently, each subgroup of NMIBC should be followed up and treated differently [3]. The main concern during treatment of NMIBC is progression to a muscle invasive stage (T2), which dramatically worsens prognosis [4]. To prevent this scenario, clinical and pathological factors are commonly used to categorize patients into different risk groups. These methods, such as the EORTC (European Organization for Research and Treatment of Cancer) Risk Tables, help physicians predict the probability of progression and recurrence, and ultimately – help decide the most appropriate treatment [3, 5].

However, these grouping systems are far from optimal: would a probability of recurrence of 35% per year justify an aggressive treatment? Is a 15% chance of progression per 1 year a sufficient reason to perform a cystectomy? [5]. Thus, we still lack a strong prognostic factor that could help predict patient-specific risk rather than group-specific risk of recurrence and progression.

According to recent studies cited below, the systemic inflammatory response state triggered by the tumor microenvironment alters acute phase reactants and hematologic components - including changes in serum neutrophil and lymphocyte counts that leads to relative neutrophilia and lymphocytopenia. This state of elevated Neutrophil-Lymphocyte ratio (NLR) is associated with worse disease-free and overall survival in a variety of different malignancies [6–8].

Among patients with bladder cancer, an elevated NLR was associated with advanced stage, increased mortality, and decreased overall survival in patients with muscle-invasive disease [9–11], along with higher risk of recurrence and progression in non-muscle invasive disease [12, 13]. Specifically, in both our retrospective studies which employed different methods of analysis, NLR > 2.5 was found to be a significant predictor of recurrence [12, 13]. Following these results, and in addition to the fact that prospective data regarding the role of NLR in predicting disease recurrence and progression in NMIBC have never been published, the aim of the current study was to prospectively evaluate the role of NLR > 2.5 as a predictor of disease recurrence in patients with primary NMIBC.

## Methods

### Study design & procedures

This was a single center, prospective cohort study. Recruited patients were pathologically confirmed to have non-invasive BC stages – Ta, T1 and Tis, after undergoing trans-urethral resection of bladder tumor (TUR-BT). Tumors were graded and staged according to the 2004 WHO grading system [14]. Pre-operative NLR was recorded using the admission’s (usually 24 h prior to surgery) complete blood count (CBC) with differential. Follow up invitations were sent out every 3 months for urine cytology, upper tract imaging, cystoscopy and treatment based on the American Urological Association (AUA) guidelines [15]. We point out that given the nature of a prospective study design, an intervention that might affect the variables is not desirable, and hence the treatment was chosen according to best practice guidelines and not according to our assumption that NLR may play a role. The end-point of the follow up was either a cancer recurrence or the termination of the study. Some degree of non-compliance to the follow up and treatment was expected, and so the last date of follow-up was recorded for missing and deceased patients. This study was based on the principles of Helsinki and was approved by the institutional review board.

### Objectives

A primary objective of the study was to evaluate the effect of NLR > 2.5 on NMIBC recurrence after trans-urethral resection of bladder tumor (TUR-BT). This effect on recurrence was to be evaluated against the current standard means to predict recurrence, which is the EORTC’s prediction table. Secondary objectives were to evaluate the effect of NLR > 2.5 on recurrence, when stratified by different variables including the pathologic grade, stage and the intra-vesical treatment. These objectives were set in advance, and were meant to test the hypothesis that a prediction of recurrence by NLR > 2.5 can be produced prospectively, and not only retrospectively [12, 13].

### Participants

Eligible Patients were ≥ 18 years with pathologically confirmed NMIBC who underwent trans-urethral resection of bladder tumor (TUR-BT) since December 2013. An Inclusive approach was taken in order to examine broad and general effect of NLR, not only on some naïve or carefully chosen groups. Key exclusion criteria were: T2 Stage, hematologic malignancies, acute infections, and patients without preoperative NLR. All pathological grades were included.

### Statistical analysis

Clinical features between groups were evaluated using Student *t*-test or chi-square test. Recurrence-free survival was evaluated using Kaplan-Meier survival plots and Log Rank was used to compare between groups. Univariate and multivariate Cox regressions were performed to assess the NLR2.5 predictive capability for recurrence, versus and in conjunction to the EORTC score. The analysis was first performed for the whole cohort, and next stratified by the ‘Treatment Type’ Groups: ‘No Treatment’, ‘Mitomycin C (MMC)’ or ‘Bacillus Calmette–Guérin (BCG)’, as the treatment choice should affect the recurrence in a meaningful way.

The EORTC Score was calculated in accordance to Sylvester et al. [5]. The tumors’ pathological variables are inherently included in the EORTC score, in a way that is already established to be statistically significant. As such, further statistical analysis of the pathological variables is redundant. The results are presented as hazard ratios along with their 95% confidence intervals. A 2-sided *P* value of < 0.05 was considered statistically significant. Data was analyzed using IBM SPSS v23.0.

## Results

Between December 2013 and October 2016, 113 patients were recruited to the study. The cohort included 96 men and 17 women with a median age of 72 years (IQR 63, 81) with a confirmed pathological diagnosis of NMIBC. Sixty-four patients (56.6%) have had a recurrence during the study, occurring at the median time of 9 months (IQR 6, 13), while the median follow-up time for patients without recurrence was 18 months (IQR 14, 30). The median NLR was 2.69 (IQR 1.9, 4.35) including 69 patients (58%) who have had NLR > 2.5. Table 1 shows an analysis of differences in clinical features between groups divided by recurrence. Table 2 shows an analysis of differences in clinical features between groups divided by NLR-2.5.

**Table 1:** Patient and tumor characteristics of the study cohort stratified by recurrence

		Patient Groups						*P*-Value
		No Recurrence			Recurrence			
		Count	Row *N* %	Median (IQR)	Count	Row N %	Median (IQR)	
Age		49	43.4%	70 (62, 78)	64	56.6%	75 (65, 83)	0.290
Sex	Female	6	35.3%		11	64.7%		0.466
	Male	43	44.8%		53	55.2%		
Grade	1	35	45.5%		42	54.5%		0.765
	2	1	50.0%		1	50.0%		
	3	13	38.2%		21	61.8%		
Stage	Ta	38	46.3%		44	53.7%		0.299
	T1	11	35.5%		20	64.5%		
CIS	No	47	42.7%		63	57.3%		0.409
	Yes	2	66.7%		1	33.3%		
Number Of Tumors	Single Tumor	14	40.0%		21	60.0%		0.885
	2–7 Tumors	29	44.6%		36	55.4%		
	8 or More	6	46.2%		7	53.8%		
Tumor Diameter	< 30 mm	36	47.4%		40	52.6%		0.218
	30 mm or more	13	35.1%		24	64.9%		
Past TCC	No	33	41.3%		47	58.8%		0.480
	Yes	16	48.5%		17	51.5%		
WBC		49	43.4%	7.9 (7.05, 9.66)	64	56.6%	7.71 (6.17, 10)	0.373
NLR		49	43.4%	2.35 (1.7, 3.43)	64	56.6%	2.87 (2.29, 4.65)	0.287
NLR-2.5	Below 2.5	28	59.6%		19	40.4%		0.004
	Above 2.5	21	31.8%		45	68.2%		
Treatment Type	No Treatment	15	34.1%		29	65.9%		0.053
	MMC	12	37.5%		20	62.5%		
	BCG	22	59.5%		15	40.5%

**Table 2:** Patient and tumor characteristics of the study cohort stratified by neutrophil-to-lymphocyte ratio (NLR)

		Patient Groups						*P*-Value
		Below 2.5			Above 2.5			
		Count	Row *N* %	Median (IQR)	Count	Row *N* %	Median (IQR)	
Age		47	41.6%	69 (59, 75)	66	58.4%	77 (70, 83)	
Status	No Recurrence	28	57.1%		21	42.9%		0.003
	Recurrence	19	29.7%		45	70.3%		
Sex	Female	6	35.3%		11	64.7%		0.568
	Male	41	42.7%		55	57.3%		
Grade	1	35	45.5%		42	54.5%		0.293
	2	0	0.0%		2	100.0%		
	3	12	35.3%		22	64.7%		
Stage	Ta	40	48.8%		42	51.2%		0.012
	T1	7	22.6%		24	77.4%		
CIS	No	44	40.0%		66	60.0%		0.037
	Yes	3	100.0%		0	0.0%		
Number Of Tumors	Single Tumor	14	40.0%		21	60.0%		0.635
	2–7 Tumors	26	40.0%		39	60.0%		
	8 or More	7	53.8%		6	46.2%		
Tumor Diameter	< 30 mm	30	39.5%		46	60.5%		0.512
	30 mm or mo re	17	45.9%		20	54.1%		
Past TCC	No	32	40.0%		48	60.0%		0.593
	Yes	15	45.5%		18	54.5%		
WBC		47	41.6%	7.58 (6.17, 8.6)	66	58.4%	8.91 (7.05, 10.5)	0.007
Treatment Type	No Treatment	17	38.6%		27	61.4%		0.317
	MMC	11	34.4%		21	65.6%		
	BCG	19	51.4%		18	48.6%		

Similar to our retrospective study, NLR (> 2.5) was correlated significantly with recurrence (*p* = 0.003) but also with age (68 vs 78 years, *P* = 0.0001) and stage (*p* = 0.01). The significant *p*-value correlation with CIS is irrelevant as only 3 patients had CIS.

**Fig. 1:**
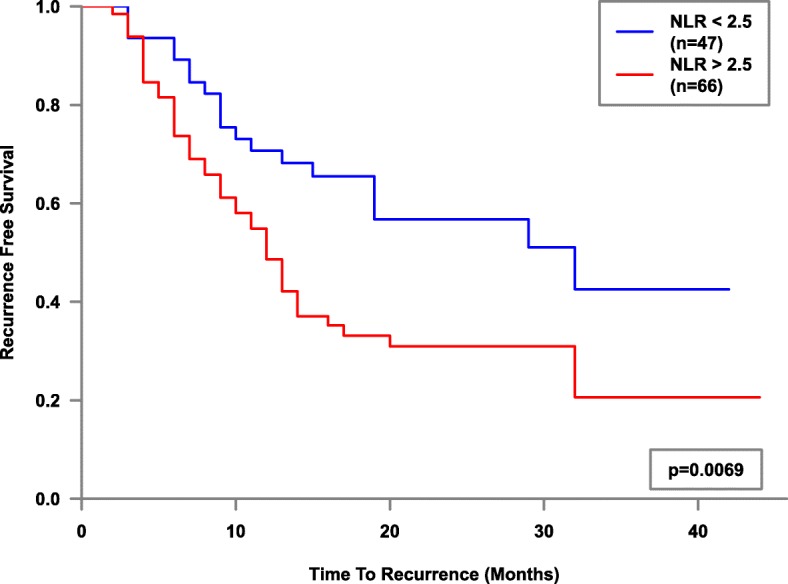
Kaplan-Meier estimates of recurrence-free survival factored by NLR 2.5 - whole cohort analysis

**Fig. 2:**
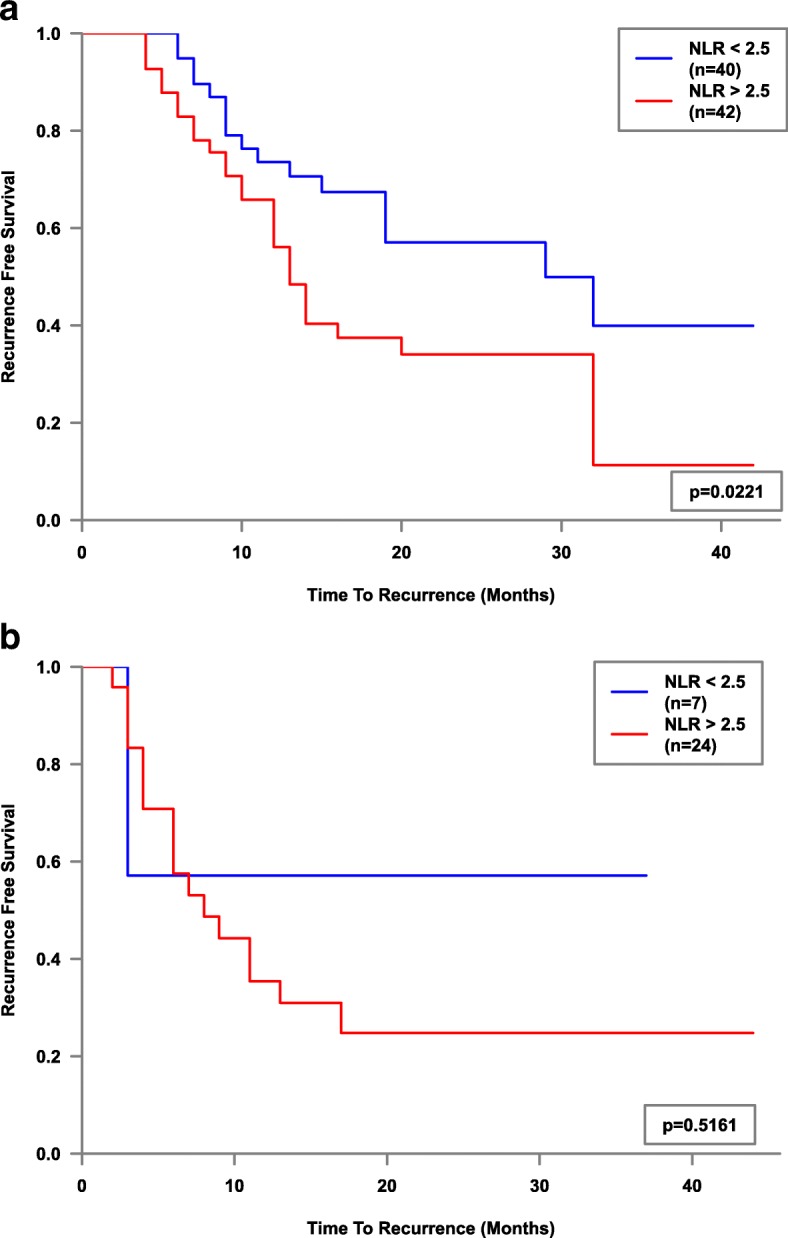
Kaplan-Meier estimates of recurrence-free survival factored by NLR 2.5 for non-muscle invasive stages Ta (**a**) and T1 (**b**)

**Fig. 3:**
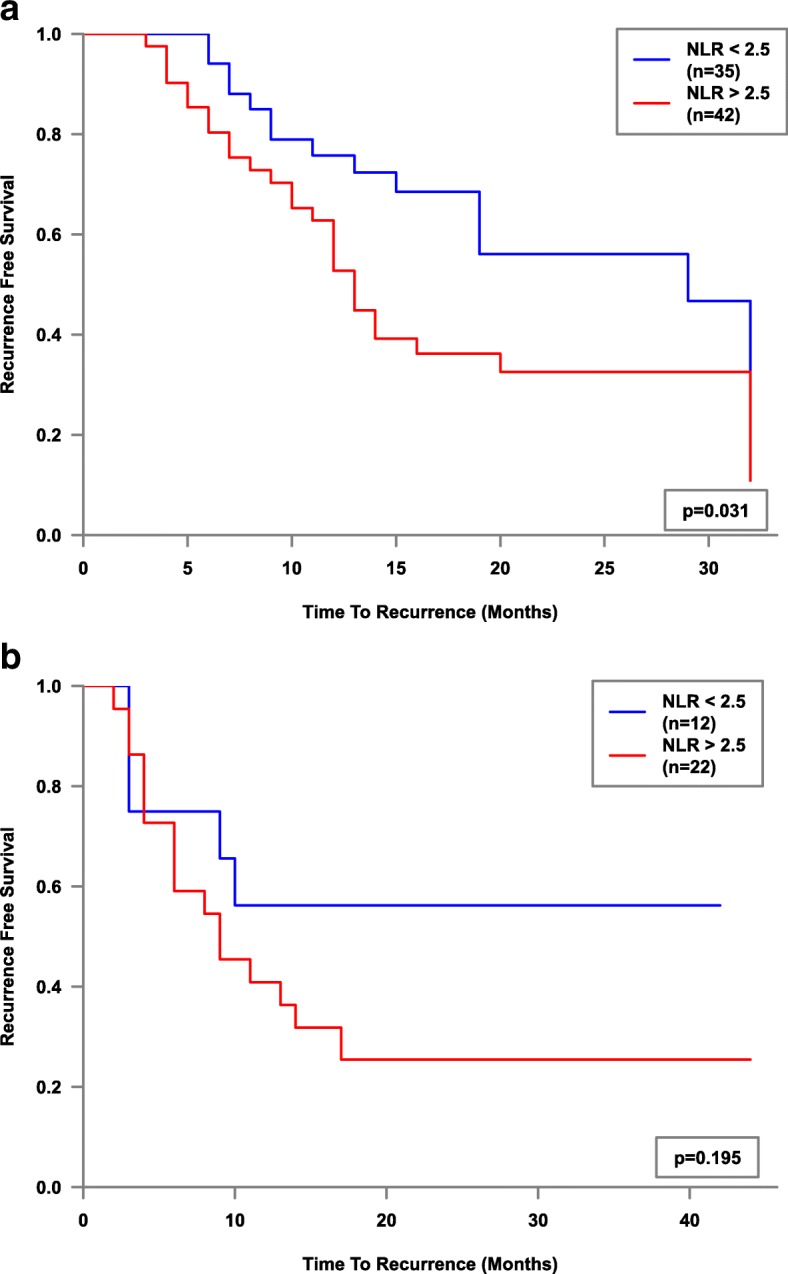
Kaplan-Meier estimates of recurrence-free survival factored by NLR 2.5 for low (**a**) and high (**b**) pathological grades

**Fig. 4:**
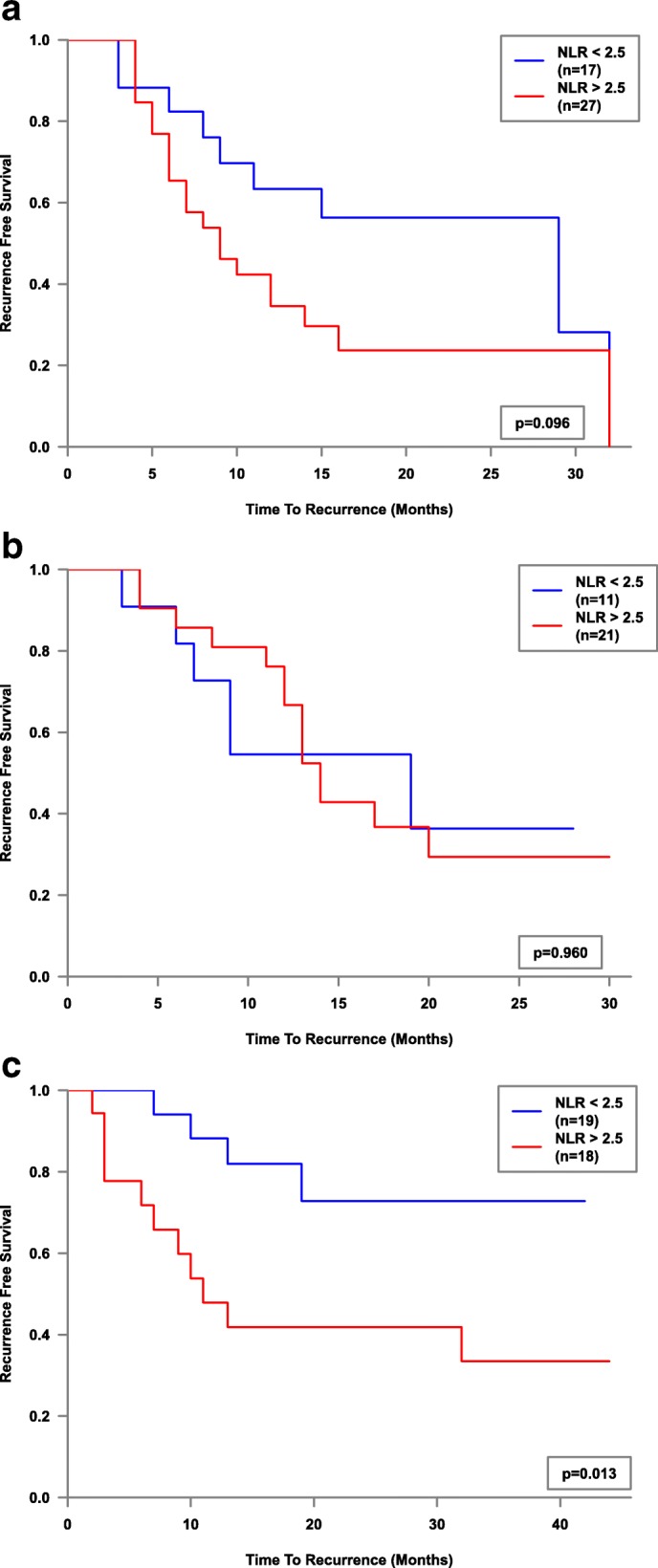
Kaplan-Meier estimates of recurrence-free survival factored by NLR 2.5 for ‘No Intravesical Treatment’ (**a**), ‘MMC’ (**b**) and ‘BCG’ (**c**) and subgroups

Whole cohort Kaplan-Meier survival plot factored by NLR2.5 was then performed and showed a significant difference (*p* = 0.007) in mean recurrence-free survival - (18.6 months vs 26.7 months, Fig. 1). Mean recurrence-free survival of NLR > 2.5 stratified by stage, grade and treatment type (sub-group analysis), showed statistical significance for the Ta Stage (*p* = 0.022, 18.7 vs 27 months), G1 Grade (*p* = 0.031, 17.1 vs 23 months) and the BCG sub-group (*p* = 0.013, 21.3 vs 34.1 months) of 37 patients, Figs. 2, 3 and 4. Sub-group breakdown (i.e Ta stage and T1 stage) is presented as lettered graphs ("A", "B" etc) under each figure. A persistent trend albeit without statistical significance was seen for the other stratifications (T1 Stage, G3 Grade and the other treatment types) in that the NLR > 2.5 groups always fared worse than the NLR < 2.5 groups.

In the univariate, whole cohort NLR2.5 Cox regression for recurrence, NLR2.5 was found significant (*p* = 0.01) with Hazard ratio of 2.029 (CI 1.185–3.472), indicating that the probability of recurrence is increased at least 2-fold for a person with NLR > 2.5 compared with NLR < 2.5 in this whole cohort analysis. After stratification by Treatment Type, NLR2.5 was only found significant for the ‘BCG’ subgroup (*p* = 0.023) with Hazard Ratio of 3.792 (CI 1.2–11.9) and not for the ‘No Treatment’ and ‘MMC’ subgroups (*p* = 0.123 and *p* = 0.96 respectively). (Table 3).

**Table 3:** Univariate Cox Regression for recurrence using the NLR 2.5 cutoff, stratified by treatment subgroups

			95.0% CI for HR	
Group	*P*-Value	Hazard Ratio	Lower	Upper
Whole Cohort	0.010	2.029	1.185	3.472
No Treatment	0.123	1.866	0.845	4.123
MMC	0.960	1.025	0.392	2.677
BCG	0.023	3.793	1.203	11.956

An identical Cox analysis was then done for the EORTC Score, which resulted in a significance only for the ‘No Treatment’ subgroup (*p* = 0.024) with Hazard ratio of 1.278 (CI 1.03–1.58), and interestingly not for the whole cohort (*p* = 0.132) or the other subgroups. (Table 4).

**Table 4:** Univariate Cox Regression for recurrence using the EORTC score, stratified by treatment subgroups

			95.0% CI for HR	
Group	*P*-Value	Hazard Ratio	Lower	Upper
Whole Cohort	0.132	1.085	0.976	1.207
No Treatment	0.024	1.278	1.033	1.580
MMC	0.266	1.128	0.913	1.393
BCG	0.934	1.008	0.841	1.207

When NLR2.5 and the EORTC Score are analyzed together as a multivariate Cox model, the results per subgroups are retained: the EORTC Score is only significant for the ‘No Treatment’ subgroup (*p* = 0.039) and NLR2.5 is only significant for the BCG subgroup (*p* = 0.025). Although in contrast to the Univariate models, EORTC is very close to significance (*p* = 0.058, HR 1.11, CI 0.996–1.241) when taken together with NLR2.5 (*p* = 0.012, HR 2.098, CI 1.174–3.75). (Table 5).

**Table 5:** Multivariate Cox Regression for recurrence using both the EORTC score and NLR 2.5 Cutoff, stratified by treatment subgroups

				95.0% CI for HR	
Group	Variable	*P*-Value	Hazard Ratio	Lower	Upper
Whole Cohort	NLR2.5	0.012	2.098	1.174	3.750
	EORTC	0.058	1.112	0.996	1.241
No Treatment	NLR2.5	0.273	1.673	0.666	4.201
	EORTC	0.039	1.267	1.012	1.587
MMC	NLR2.5	0.640	1.285	0.449	3.682
	EORTC	0.233	1.138	0.920	1.408
BCG	NLR2.5	0.025	3.962	1.193	13.159
	EORTC	0.396	1.086	0.898	1.312

## Discussion

The main advantage of this study is its prospective nature, which to our knowledge, is one of the firsts to deal with NLR as a predictor for NMIBC. Upon diagnosis, NMIBC is initially treated with complete TUR-BT, after which an adjuvant therapy is considered. Based on clinical and pathological factors, patients can be assigned to risk groups, such as the EORTC Score for the assessment of disease recurrence and progression [5]. However, these predictive tools are far from optimal for the individual patient – what is the progression probability cutoff that justifies cystectomy? How aggressive an intra-vesical treatment should be with a 35% risk of recurrence per year? To be able to answer these kinds of questions in a more evidence-based manner, new and novel predictors are a necessity.

In the current study, we prospectively assessed the predictive value of NLR versus and in conjunction to the EORTC score in a group of NMIBC patients. The first main finding for the whole cohort include a statistically significant association between high NLR (> 2.5) and increased probability of recurrence – a finding that manifests in shorter time to recurrence.

In addition, high NLR was consistently associated with worse outcomes in all the sub-groups, although significance was demonstrated only for the Ta Stage, G1 Grade and the BCG treatment group. We believe that given a larger cohort per sub-group, a statistical significance is probable. Nevertheless, the trend is clear – patients with higher NLR presented with worse recurrence-free survival in each stratification. NLR ratio was more significant in patients who received BCG compare to those who received MMC. We may assume that as an immune modulator BCG has better effect in patients with lower NLR. As this is a new finding arising from a prospective study our aim is to keep on analyzing this subgroup in our next prospective study.

The second main finding is the apparent synergistic effect between NLR (> 2.5) and the EORTC score, as the significance of the score increased substantially when calculated alongside the NLR2.5 variable. The EORTC score was used as a measure of reference, as it has been already established for thousands of patients. However, the EORTC score was never designed to be used when BCG intra-vesical treatment is chosen, as was clearly stated in reference [5]. This limitation of the EORTC score matches our results, as this score is undoubtedly significant for the group that received no treatment, but insignificant for whole cohort which includes the BCG treated patients. Luckily, the NLR > 2.5 is specifically significant for the BCG subgroup, in a manner that complements the EORTC score and improves the overall prediction for the whole-cohort.

While the pathophysiology is not yet clear, it has been suggested that the relative neutrophilia increases the number of inflammatory markers that include pro-angiogenic factors (VEGF), growth factors (CXCL8), proteases and anti-apoptotic markers (NF-kB) – all of which support tumor growth and progression. In addition, the lymphocytopenia is suggested to hurt cell-mediated immune response and thus worsening prognosis [16].

Pretreatment NLR is readily available, and higher values have been shown to correlate with higher stage tumors and adverse treatment outcomes in a wide variety of cancers including malignancies of the gastrointestinal and genitourinary tracts, including urothelial carcinoma of the bladder [6, 7, 12, 13].

Focusing on bladder cancer, several previous studies have evaluated the predictive value of NLR, most of which were conducted on patients undergoing radical cystectomy [9, 17–19]. Based on these studies, NLR may be used in the pre-operative setting to predict tumor invasiveness, or in the post-operative setting, together with pathologic tumor characteristics, to predict outcome. Can et al. found a correlation between muscle invasive disease in TURBT specimens and preoperative NLR > 2.57, patient age, female gender and platelet count, and suggested using NLR > 2.57 in a risk formula which may assist in deciding which patients may benefit from early cystectomy [17]. Similarly, Krane et al. found that patients with a NLR > 2.5 had a significantly higher likelihood of extravesical disease at radical cystectomy, suggesting that they may benefit from neoadjuvant chemotherapy [10]. Finally, Viers et al. found an association between higher pre-operative NLR and significantly increased risk of extravesical tumor extension and lymph node involvement, in a large group of bladder cancer patients undergoing radical cystectomy [9].

Curiously, most of the studies investigating the role of NLR in patients with NMIBC specifically have been retrospective – including our own two previously published articles [12, 13]. To date, only two prospective studies on the matter have been published, after this study’s initiation. Favilla et al. further established the predictive value of NLR on recurrence, but did not elaborate regarding the relationship between NLR and the EORTC score [20]. Sebahattin et al. argued that correction for age might alter the results, so a logistic regression analysis (backwards, conditional) of the NLR2.5 and Age as a covariate, was performed. This regression resulted in only NLR2.5 as a significant variable (*p* = 0.005) with Odds ratio of 3.045 (CI 1.392–6.661), meaning that there is an average of at least 3-fold higher probability of recurrence for a person with NLR > 2.5 compared with NLR < 2.5. Age was removed from the model because of insignificance (*p* = 0.988) [21].

### Limitations

A prominent limitation dealing with the NLR marker is the volatility of the Neutrophil and Lymphocyte counts. While we did actively exclude patients with hematologic malignances and with active infections, it is possible that some chronic medications or antibiotics affect the NLR value. An argument can be made that this approach might skew results, but as mentioned in the ‘Materials’ section – we strived to examine the effect of NLR on as much patients as possible, with the intention to generalize, and not marginalize, the NLR usability. We believe that the inclusive cohort in this study (i.e. including a small number of possible antibiotic users) can be regarded more like hurdle rather than a helpful measure, and thus the results are more meaningful. Evidence to this claim can be found on our previous publication, which dealt with a much more ‘distilled’ cohort [13].

Another limitation of the study is the small cohort per different subgroups. This has resulted in a discrepancy between the literature and our data regarding the known incidence rates of concomitant CIS. A possible explanation can either be attributed to chance, or the notion that many patients with concomitant CIS are discovered already in T2 stage, and thus were not included in this study.

We believe that given a larger cohort per sub-groups such as treatment type or pathological stage, a statistical significance is probable. A larger prospective study may be required to further solidify the place of NLR in predicting disease recurrence in patients with NMIBC and to incorporate it in the current risk calculation tools.

## Conclusions

NLR > 2.5 was found to be a significant predictor of disease recurrence and demonstrated high hazard ratio and worse recurrence-free survival in patients with NMIBC, especially in those treated with BCG. Additionally, our data demonstrated statistical evidence that NLR > 2.5 might have an improving effect on the EORTC score’s prediction when calculated together. Thus, we propose to consider the incorporation of NLR > 2.5 in the next revisions of the EORTC score.

## Abbreviations

AUA: American Urological Association
BCG: Bacillus Calmette–Guérin
CBC: Complete blood count
EORTC: European Organization for Research and Treatment of Cancer
MMC: Mitomycin C
NLR: Neutrophil-to-lymphocyte ratio
NMIBC: Non-muscle invasive bladder cancer
TUR-BT: Trans-urethral resection of bladder tumor
